# Multiparametric models for predicting major arrhythmic events in Brugada syndrome: a systematic review and critical appraisal

**DOI:** 10.1093/europace/euaf091

**Published:** 2025-05-02

**Authors:** Daniel A Gomes, Pier D Lambiase, Richard J Schilling, Riccardo Cappato, Pedro Adragão, Rui Providência

**Affiliations:** Department of Cardiology, Hospital de Santa Cruz, Lisbon, Portugal; Institute of Cardiovascular Science, University College London, London, UK; Barts Heart Centre, St Bartholomew’s Hospital, Barts Health NHS Trust, London, UK; Barts Heart Centre, St Bartholomew’s Hospital, Barts Health NHS Trust, London, UK; William Harvey Research Institute, Faculty of Medicine and Dentistry, Queen Mary University of London, London, UK; Arrhythmia and Clinical Electrophysiology Center, IRCCS, MultiMedica, Milan, Italy; Department of Cardiology, Hospital de Santa Cruz, Lisbon, Portugal; Department of Cardiology, Hospital da Luz, Lisbon, Portugal; Barts Heart Centre, St Bartholomew’s Hospital, Barts Health NHS Trust, London, UK; Institute of Health Informatics Research, University College London, 222 Euston Road, London NW1 2DA, UK

**Keywords:** Brugada syndrome, Sudden cardiac death, Ventricular arrhythmia, Risk scores, PROBAST, Methods

## Abstract

**Aims:**

Despite several risk models to predict major arrhythmic events (MAE) in Brugada syndrome (BrS) having been developed, reproducibility and methodology remain a concern. Our aim was to assess the quality of model development and validation, and determine the discriminative performance of available models.

**Methods and results:**

Electronic databases (Medline, Embase, and Central) were searched through September/2024 for studies developing or validating multivariable prediction models for MAE in BrS. Methodological quality and risk of bias (RoB) were assessed using the Critical Appraisal and Data Extraction for Systematic Reviews of Prediction Modelling Studies (CHARMS) checklist and the Prediction Model Risk of Bias Assessment (PROBAST) Tool. Pooled random-effects c-statistics were obtained for each model. A total of 16 studies, including 11 unique multivariable scores, were included. All models had domains classified as high RoB. Common sources of bias were inappropriate inclusion/exclusion criteria, predictor selection, low number of events and underreporting of performance measures. Pooled c-statistics among patients without previous MAE showed good performance for Brugada-Risk [AUC 0.81, 95% confidence interval (CI) 0.71–0.91; *I*^2^ 64%; three studies], fair for PAT (AUC 0.79, 95% CI 0.45–1.12; *I*^2^ 95%; two studies), Delise (AUC 0.77, 95% CI 0.72–0.81, *I*^2^ 39%, three studies), and Sieira (AUC 0.73, 95% CI 0.64–0.82; *I*^2^ 64%; five studies), and moderate for Shanghai (AUC 0.69, 95% CI 0.61–0,76; *I*^2^ 13%; three studies).

**Conclusion:**

Currently available multiparametric models for prediction of MAE in BrS have important shortcomings in model development and inadequate evaluation. Further validation of current models in external cohorts is required before safe transition to clinical practice.

## Graphical Abstract

**Graphical Abstract:**
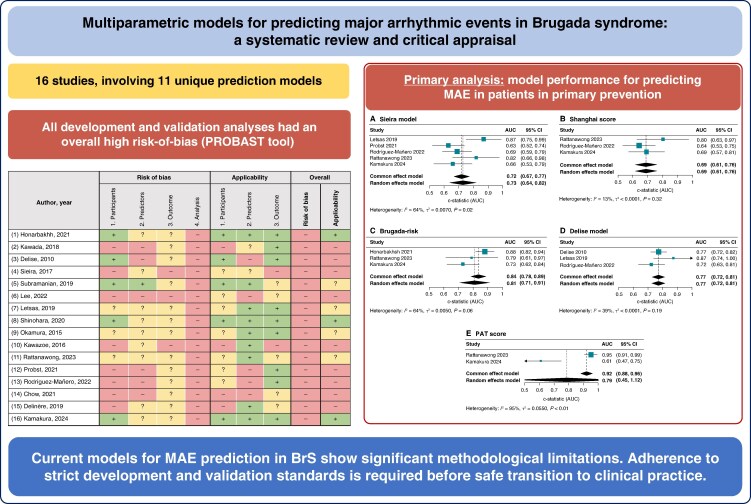

## Introduction

Brugada syndrome (BrS), first described as a clinical entity in 1992, is an inherited cardiac channelopathy characterized by atypical right bundle branch block with coved ST-segment elevation (>2 mm) in the right precordial leads (Type 1 pattern) and increased risk of ventricular tachycardia (VT), ventricular fibrillation (VF), and sudden cardiac death (SCD).^1^ This pattern arises in the context of a structurally normal heart and can occur either spontaneously or be induced by fever or exposure to sodium channel-blocking drugs.^1,2^ According to current guidelines, BrS is diagnosed in patients presenting either with a spontaneous type 1 pattern or with a drug-induced type 1 along with additional clinical features, (including history of arrhythmic syncope, VT/VF, aborted SCD, family history of BrS, or arrhythmic SCD), provided phenocopies have been excluded.^3^

Despite most patients being asymptomatic at diagnosis, in some series up to 10% may develop life-threatening ventricular arrhythmias or sudden death during follow-up periods of up to 10 years.^4–6^

Major arrhythmic events (MAE) in patients with BrS include SCD, sustained VT/VF, and appropriate implantable cardioverter-defibrillators (ICD) shocks. Identifying patients at risk for MAE is of utmost importance for preventing these ominous outcomes. Although ICD indication is well defined as a Class I recommendation in secondary prevention of SCD, risk stratification is particularly challenging in asymptomatic patients due to low event rates and conflicting evidence, with no single variable able to accurately predict arrhythmic events.^3,5–7^

A multivariable prediction model for MAE in BrS, providing accurate risk estimates for individual patients, would allow for the better selection of appropriate candidates for ICD in primary prevention. Over the past years, numerous models have been proposed to address this question.^8–11^ However, concerns remain regarding their development, which is based on relatively small retrospective studies with low number of events and limited validation in external cohorts.^12^ This is, in fact, similar to other inherited cardiac conditions such as idiopathic VF, early repolarization syndrome and long QT, where temporal and geographical variations in syndrome definitions, low incidence in the general population, and low event rates, even among high-risk patients, have made many risk models prone to significant bias and unsuitable for routine use.

Previous systematic reviews on BrS addressed this issue but were limited by either a lack or minimal critical appraisal of development methodology, or by a non-comprehensive bibliographic database search strategy.^12,13^ The aim of this systematic review of studies describing the development or external validation of multiparametric models for predicting MAE in BrS patients was to (i) critically appraise the methodology utilized in model development, and assessing the risk of bias and (ii) provide a comprehensive assessment of the discriminative power of each individual model in predicting MAE.

## Methods

This systematic review was conducted in accordance with the Preferred Reported Items for Systematic Reviews and Meta-analyses (PRISMA) guidelines.^14^ The protocol was registered in the international prospective register of systematic reviews (PROSPERO, CRD42024586543).

### Search strategy and selection criteria

We performed a systematic search of three electronic databases (MEDLINE, EMBASE, and CENTRAL) 16/09/2024, using (but not limited) the following key search words: ‘Brugada syndrome’, ‘prediction’, ‘risk model’, ‘score’, ‘sudden cardiac death’, ‘ventricular fibrillation’, and ‘ventricular tachycardia’. The full search strategy is presented in Supplementary material online, *Table S1*. Reference lists of potentially eligible studies were searched for additional sources of information. No start date or language restrictions were applied. Studies were considered suitable if they (i) developed or externally validated a clinical multiparametric score to predict MAE in BrS patients, as defined by the occurrence of SCD, sustained VT/VF, or appropriate ICD shocks and (ii) included adult (≥18 years old) patients. Only full-text articles were included, and studies developing models based on artificial intelligence (AI) alone were not considered due to lack of traceability of the predictors used (i.e. models with no clear information on included variables and their weighting). Two investigators (D.G. and R.P.) independently screened and selected potentially eligible studies based on title and abstract. Final eligibility was decided after evaluation of full-text publications. All disagreements were resolved via discussion or through the involvement of a third referee (P.A.).

### Data extraction and quality assessment

Data extraction was undertaken independently by two investigators (D.G. and R.P.) based on the Critical Appraisal and Data Extraction for Systematic Reviews of Prediction Modelling Studies checklist (CHARMS), and all disagreements were resolved via discussion, or through the involvement of a third referee (P.A.).^15^ A standardized form was used to extract the following information from each study: (i) study design, number of events, outcome definition, and follow-up; (ii) baseline characteristics of the participants (age, sex, spontaneous Brugada pattern type 1, prior syncope, family history of SCD, previous aborted SCD or ventricular arrhythmia, genetic testing, electrophysiological study); (iii) information on predictors and model development; and (iv) information on model discrimination, calibration and validation (see Supplementary material online, *Table S2*).^16^

Following data extraction, a pair of reviewers (D.G. and R.P.) independently assessed study methodological quality according to the Prediction Model Risk of Bias Assessment Tool (PROBAST).^17^ PROBAST is a tool designed to assess the risk of bias and concerns about applicability of studies in which a multiparametric diagnostic or prognostic model is developed or validated. The assessor evaluates the risk of bias based on four domains of signalling questions: (i) the number and characteristics of participants, (ii) the definition of predictors, (iii) the definition and assessment of outcomes, and (iv) model analysis and performance measures (see Supplementary material online, *Table S2*).^17^ Models scoring ‘high risk of bias’ in at least one domain were considered as overall high risk. All disagreements were resolved via discussion, or through the involvement a third referee (P.A.).

### Data synthesis and analysis

Data were synthesized if reported in at least two included studies. Continuous variables were presented as mean and standard deviation (SD) or medians and interquartile range (IQR), when appropriate. The outcome of interest was MAE, as previously defined. Pooled discriminative power of each model, as assessed by the reported c-statistics (AUC) and 95% confidence intervals (95% CI), were used as summary statistics and were calculated using both the DerSimonian and Laird random-effects and fixed-effects model. When the 95% CI were not reported, we estimated them based on the size of the population and number of events.^18^ Heterogeneity across studies was assessed by *I*^2^ using Cochran’s Q test, where values of less than 25, 50, and 75% were regarded as evidence of low, moderate, and high levels of heterogeneity, respectively.^19^ Pooled c-statistics values between 1 and 0.9 were considered ‘excellent’, 0.9 and 0.8 ‘good’, 0.8 and 0.7 ‘fair’, 0.7 and 0.6 ‘poor’, and 0.6–0.5 ‘failed’.

The primary analysis was individual model performance for predicting MAE in patients in primary prevention (i.e. without prior VT/VF or aborted SCD), as there is consensus on the indication for secondary prevention ICD in BrS (Class of recommendation I, level of evidence C).^3^ Studies included in this analysis were those that either excluded patients with prior VF/aborted SCD from the entire derivation or validation cohort, or provided subgroup analyses that omitted these patients. A subsequent analysis including results for both primary and secondary prevention BrS was performed. Analyses were undertaken using the R version 4.3.2 (packages: ‘meta’, ‘ggplot2’, and ‘shiny’).

## Results

### Study selection and characteristics

A total of 3651 records were identified through database searching. Following exclusion of duplicates and screening, 31 studies were identified for full-text review, and further 15 were subsequently excluded (see Supplementary material online, *Table S3*).

**Figure 1:**
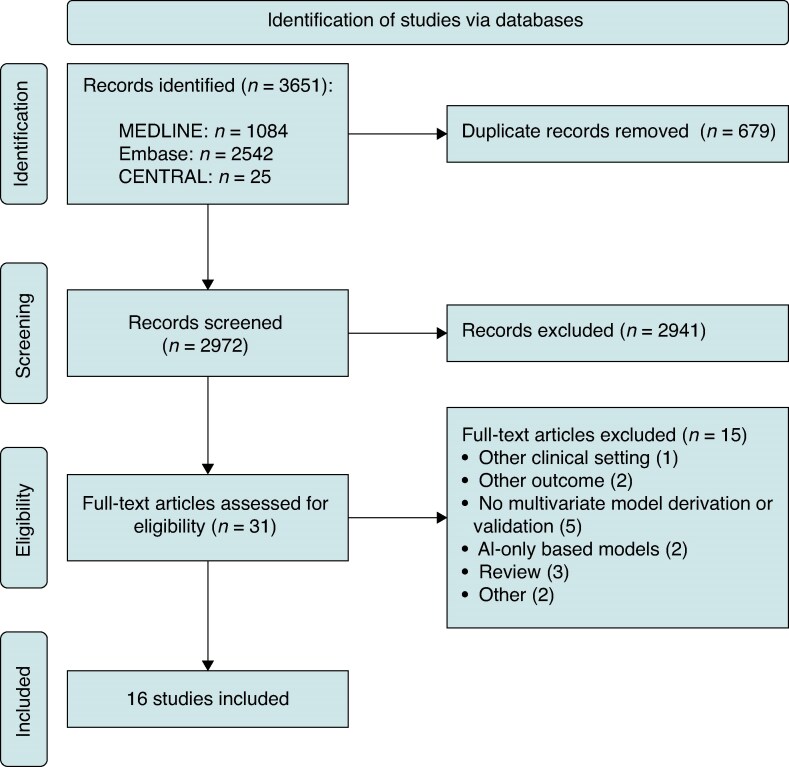
PRISMA flowchart.

Overall, 16 different records published between 2010 and 2024 were included in the final analysis (*Figure 1*).^8–11,20–31^ There was full agreement between investigators (D.G. and R.P.) on the eligibility of the selected studies. A summary of the main characteristics is presented in *Table 1*. Briefly, study types included model development, alone (*n* = 4, 25.0%) or with external validation of the new and/or existing models (*n* = 6, 37.5%), and external validation only (*n* = 6, 37.5%).

**Table 1:** Study, model, and analysis details for included studies

Reference	Analysis type (distinguishing feature of model from others developed in same study)	Derivation model name	For external validations, reference for model development study if different from listed study (model name, if applicable)	Region	Years	Number of patients	Outcome	Follow-up	Number of events (%)
(1) Honarbakhh (2021)	D + EV	BRUGADA-RISK		International	Unclear	1110	VA/SCD	5.3 ± 4.0 years	114 (10.3)
(2) Kawada (2018)	EV		Shanghai score	Japan	1996–2016	393	VA/SCD	97.7 (39.7–142.1) months	43 (10.9)
(3) Delise (2010)	D	Delise model		Italy	1998–2009	320	VA/SCD	40 (20–67) months	17 (5.3)
(4) Sieira (2017)	D + EV	Sieira model		Belgium	1992–2013	400	VA/SCD	80.7 ± 57.2 months	34 (8.5)
(5) Subramanian (2019)	D + EV	Subramanian model		India	2007–2016	103	VA/SCD	85.3 (55.7–97.1) months	13 (12.6)
	EV		Kawazoe model	India	2007–2016	103	VA/SCD	85.3 (55.7–97.1) months	13 (12.6)
(6) Lee (2022)	EV		Sieira model	Hong Kong	1997–2020	548	VA	84 ± 55 months	66 (12.0)
	EV		Delise model	Hong Kong	1997–2020	548	VA	84 ± 55 months	66 (12.0)
	EV		Shanghai score	Hong Kong	1997–2020	548	VA	84 ± 55 months	66 (12.0)
	EV		Okamura model	Hong Kong	1997–2020	548	VA	84 ± 55 months	66 (12.0)
	EV		BRUGAGA-RISK score	Hong Kong	1997–2020	548	VA	84 ± 55 months	66 (12.0)
	EV		Letsas model	Hong Kong	1997–2020	548	VA	84 ± 55 months	66 (12.0)
(7) Letsas (2019)	D	Letsas model		Greece	Unclear	111	VA/SCD	4.6 ± 3.5 years	7 (6.3)
	EV		Sieira model	Greece	Unclear	111	VA/SCD	4.6 ± 3.5 years	7 (6.3)
	EV		Delise model	Greece	Unclear	111	VA/SCD	4.6 ± 3.5 years	7 (6.3)
	EV		Okamura model	Greece	Unclear	111	VA/SCD	4.6 ± 3.5 years	7 (6.3)
(8) Shinohara (2020)	D	Shinohara model		Japan	2002–2015	193	VA/SCD	101 ± 48 months	7 (3.6)
(9) Okamura (2015)	D	Okamura model		Japan	1996–2012	218	VA/SCD	78 ± 49 months	26 (11.9)
(10) Kawazoe (2016)	D + IV	Kawazoe model		Japan	2001–2014	143	VA	82.8 ± 49.0 months	25 (17.5)
(11) Rattanawong (2023)	D + IV	PAT score		International	1998–2020	105	VA/SCD	8.0 ± 4.1 y	60 (51.1)
	EV		PAT score	International	1998–2020	164	VA/SCD	8.0 ± 6.9 y	15 (9.1)
	EV		Shanghai score	International	1998–2020	164	VA/SCD	8.0 ± 6.9 y	15 (9.1)
	EV		BRUGAGA-RISK score	International	1998–2020	164	VA/SCD	8.0 ± 6.9 y	15 (9.1)
	EV		Sieira model	International	1998–2020	164	VA/SCD	8.0 ± 6.9 y	15 (9.1)
(12) Probst (2021)	EV		Shanghai score	International	1993–2016	1613	VA/SCD	6.5 ± 4.7 y	75 (4.6)
	EV		Sieira model	International	1993–2016	461	VA/SCD	9.4 ± 4.1 y	27 (5.9)
(13) Rodríguez-Mañero (2022)	EV		Shanghai score	International	1998–2020	831	VA/SCD	10.2 ± 4.7 years	47 (5.7)
	EV		Sieira model	International	1998–2020	831	VA/SCD	10.2 ± 4.7 years	47 (5.7)
	EV		Delise model	International	1998–2020	831	VA/SCD	10.2 ± 4.7 years	47 (5.7)
(14) Chow (2021)	EV		Sieira model	UK	2004–2019	192	VA/SCD	5.1 ± 2.8 y	22 (11.4)
(15) Delinière (2019)	D			International	Unclear	115	VA/SCD	NR	45 (39.1)
(16) Kamakura (2024)	EV		PAT score	Japan	2002–2015	413	VA/SCD	106.8 ± 66.1 months	54 (13.1)
	EV		BRUGAGA-RISK score	Japan	2002–2015	413	VA/SCD	106.8 ± 66.1 months	54 (13.1)
	EV		Shanghai score	Japan	2002–2015	413	VA/SCD	106.8 ± 66.1 months	54 (13.1)
	EV		Sieira model	Japan	2002–2015	413	VA/SCD	106.8 ± 66.1 months	54 (13.1)

Values reported as numbers and percentages, mean ± standard deviation or median (interquartile range), as appropriate.

D, derivation; EV, external validation; IV, internal validation; NR, not reported; SCD, sudden cardiac death; VA, ventricular arrhythmia.

Among the included studies, a total of 11 unique multivariable prediction models for MAE in BrS were either developed and/or externally validated. In all cases, data derived from existing registries or retrospective cohorts including patients from 1992 up to 2020. The number of participants in each study varied from 103 to 1613 (median 269) and the rate of events ranged from 3.6% to 51.1% during a mean follow-up of 3.3 to 10.2 years. The mean patient age was 41–52 years, and 58–99% were males. The incidence of spontaneous type 1 pattern was 13–81%, and 12–58% had history of syncope except in one study in which all symptomatic patients were excluded.^23^ Electrophysiological study and genetic testing were not performed routinely in most reports. Patients with a history of VT/VF or aborted SCD were excluded from 6 out of the 16 (37.5%) of the derivation and/or external validation cohorts.^8,22–25,28^ The baseline patients’ characteristics are depicted in *Table 2*.

**Table 2:** Baseline characteristics of the patients included

Reference	Analysis type (distinguishing feature of model from others developed in same study)	For external validations, reference for model development study if different from listed study	Age	Male sex	Prior syncope	Family history of SCD	Spontaneous Brugada type 1	Positive EPS study	Positive genetic testing	Previous aborted SCD/VF
(1) Honarbakhh (2021)	D + EV		51.8 ± 13.6	790 (71.2)	204 (58.0)	235 (21.2)	388 (35.0)	128/402 (31.8)	175/731 (23.9)	0
(2) Kawada (2018)	EV	Shanghai score	45 (36–56)	374 (95.2)	99 (25.2)	51 (13.0)	311 (79.1)	NR	22/167 (13.2)	23 (5.9)
(3) Delise (2010)	D		43 (33–54)	258 (81)	105 (34)	94 (29)	174 (54)	96/245 (39.2)	NR	0
(4) Sieira (2017)	D + EV		41.1 ± 17.8	233 (58.3)	111 (27.8)	184 (46.0)	78 (19.5)	72/365 (19.7)	53/215 (25.5)	20 (5.0)
(5) Subramanian (2019)	D + EV		48 (35–60)	89 (86.4)	12 (11.7)	33 (32.0)	67 (65.0)	22/53 (41.5)	NR	0
	EV	Kawazoe model	48 (35–60)	89 (86.4)	12 (11.7)	33 (32.0)	67 (65.0)	22/53 (41.5)	NR	0
(6) Lee (2022)	EV	Sieira model	49.9 ± 16.3	508 (92.7)	231 (43.3)	45 (8.2)	340 (62.0)	76/112 (67.9)	17/52 (32.7)	43 (7.9)
	EV	Delise model	49.9 ± 16.3	508 (92.7)	231 (43.3)	45 (8.2)	340 (62.0)	76/112 (67.9)	17/52 (32.7)	43 (7.9)
	EV	Shanghai score	49.9 ± 16.3	508 (92.7)	231 (43.3)	45 (8.2)	340 (62.0)	76/112 (67.9)	17/52 (32.7)	43 (7.9)
	EV	Okamura model	49.9 ± 16.3	508 (92.7)	231 (43.3)	45 (8.2)	340 (62.0)	76/112 (67.9)	17/52 (32.7)	43 (7.9)
	EV	BRUGAGA-RISK score	49.9 ± 16.3	508 (92.7)	231 (43.3)	45 (8.2)	340 (62.0)	76/112 (67.9)	17/52 (32.7)	43 (7.9)
	EV	Letsas model	49.9 ± 16.3	508 (92.7)	231 (43.3)	45 (8.2)	340 (62.0)	76/112 (67.9)	17/52 (32.7)	43 (7.9)
(7) Letsas (2019)	D		45.3 ± 13.3	86 (77.5)	37 (33.3)	7 (6.3)	49 (44.1)	32/59 (54.2)	NR	0
	EV	Sieira model	45.3 ± 13.3	86 (77.5)	37 (33.3)	7 (6.3)	49 (44.1)	32/59 (54.2)	NR	0
	EV	Delise model	45.3 ± 13.3	86 (77.5)	37 (33.3)	7 (6.3)	49 (44.1)	32/59 (54.2)	NR	0
	EV	Okamura model	45.3 ± 13.3	86 (77.5)	37 (33.3)	7 (6.3)	49 (44.1)	32/59 (54.2)	NR	0
(8) Shinohara (2020)	D		50 ± 13	180 (93)	0	67 (35)	155 (80)	51 (26.4)	NR	0
(9) Okamura (2015)	D		46 ± 13	211 (96)	87 (40)	64 (29)	159 (73)	61 (28.0)	NR	0
(10) Kawazoe (2016)	D + IV		46 ± 12	140 (97.9)	24 (16.8)	36 (25.2)	84 (58.7)	NR	NR	35 (24.5)
(11) Rattanawong (2023)	D + IV		52.2 ± 12.0	104 (99.0)	29 (27.6)	17 (16.2)	85 (81.0)	0/0	0/0	71 (67.7)
	EV	PAT score	46.5 ± 14.3	132 (80.5)	30 (18.3)	22 (13.4)	106 (64.6)	18/29 (62.1)	17/44 (38.6)	11 (6.7)
	EV	Shanghai score	46.5 ± 14.3	132 (80.5)	30 (18.3)	22 (13.4)	106 (64.6)	18/29 (62.1)	17/44 (38.6)	11 (6.7)
	EV	BRUGAGA-RISK score	46.5 ± 14.3	132 (80.5)	30 (18.3)	22 (13.4)	106 (64.6)	18/29 (62.1)	17/44 (38.6)	11 (6.7)
	EV	Sieira model	46.5 ± 14.3	132 (80.5)	30 (18.3)	22 (13.4)	106 (64.6)	18/29 (62.1)	17/44 (38.6)	11 (6.7)
(12) Probst (2021)	EV	Shanghai score	45 ± 15	1119 (69.4)	421 (26.1)	492 (30.5)	484 (30.0)	214/569 (37.6)	355 (22)	66 (4.1)
	EV	Sieira model	44 ± 13	356 (77.2)	110 (23.9)	16 (3.5)	183 (39.7)	169/461 (36.7)	NR	NR
(13) Rodríguez-Mañero (2022)	EV	Shanghai score	42.8 ± 13.1	623 (75.0)	127 (15.3)	282 (33.9)	386 (46.5)	383 (46.1)	126/390 (32.3)	28 (3.4)
	EV	Sieira model	42.8 ± 13.1	623 (75.0)	127 (15.3)	282 (33.9)	386 (46.5)	383 (46.1)	126/390 (32.3)	28 (3.4)
	EV	Delise model	42.8 ± 13.1	623 (75.0)	127 (15.3)	282 (33.9)	386 (46.5)	383 (46.1)	126/390 (32.3)	28 (3.4)
(14) Chow (2021)	EV	Sieira model	44.1 (NR)	112 (58.3)	85 (44.2)	63 (32.8)	24 (12.5)	19/88 (21.6)	NR	22 (11.4)
(15) Delinière (2019)	D		45.1 ± 12.8	105 (91.3)	42/100 (42.0)	21/80 (26.3)	115 (100.0)	31/57 (54.4)	10/57 (17.5)	NR
(16) Kamakura (2024)	EV	PAT score	50.9 ± 13.6	395 (95.6)	97 (23.5)	91 (22.0	284 (68.8)	240/324 (74.1)	0/0	99 (24.0)
	EV	BRUGAGA-RISK score	50.9 ± 13.6	395 (95.6)	97 (23.5)	91 (22.0	284 (68.8)	240/324 (74.1)	0/0	99 (24.0)
	EV	Shanghai score	50.9 ± 13.6	395 (95.6)	97 (23.5)	91 (22.0	284 (68.8)	240/324 (74.1)	0/0	99 (24.0)
	EV	Sieira model	50.9 ± 13.6	395 (95.6)	97 (23.5)	91 (22.0	284 (68.8)	240/324 (74.1)	0/0	99 (24.0)

Values reported as numbers and percentages, mean ± SD or median (IQR), as appropriate.

D, derivation; EV, external validation; IV, internal validation; NR, not reported; SCD, sudden cardiac death; VA, ventricular arrhythmia, VF, ventricular fibrillation;

### Model appraisal—PROBAST

**Figure 2:**
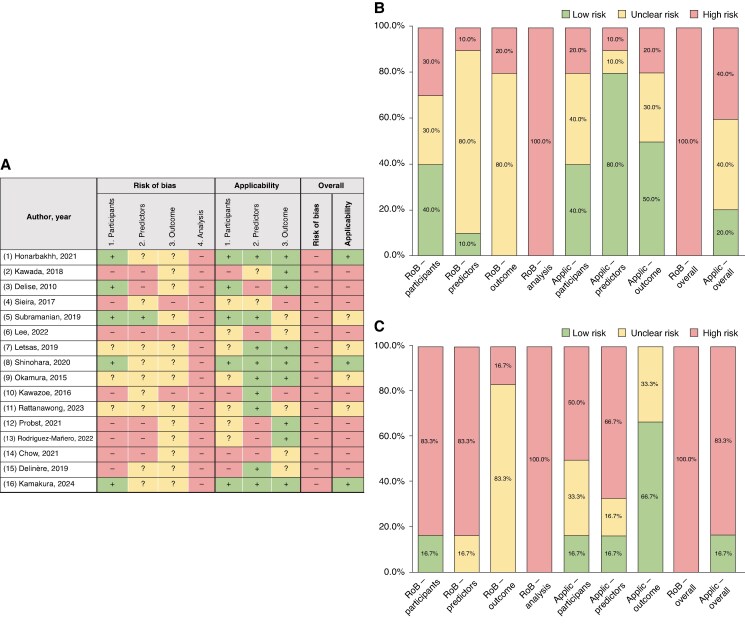
PROBAST risk of bias and applicability assessment. (*A*) Individual risk of bias and applicability assessment; (*B*) Overall PROBAST assessment in model development analyses; (*C*) Overall PROBAST assessment in external validation studies.

For the ‘participants’ domain, 3 of 10 model development analyses (30.0%) and 5 (83.3%) of external validation studies were evaluated as high risk of bias (*Figure 2*). The main reasons for the high-risk rating were the inclusion of patients with the outcome of interest at baseline (past history of VT/VF or aborted SCD),^9,10,20,21,26,29^ and use of a non-nested case–control study design (in one development and two external validation analyses).^27,29,30^

The candidate predictors and those included in the final risk scores are detailed in Supplementary material online, *Tables S4* and *S5*. The most frequent final predictors were history of syncope, spontaneous type 1 Brugada pattern, family history of SCD, early repolarization pattern in inferolateral/peripheral leads, T-peak to T-end duration ≥ 100 ms, and inducible VT/VF on electrophysiology study (EPS). Most of studies (90.0% of the development and all of the external validation analyses) were rated as unclear or high risk of bias (*Figure 2*). The main reasons for this classification were the absence of information regarding blinding for variables assessment,^8,9,11,23–25,27,30,31^ and the lack of a consistent predictor definition.^10,20,21,26,29^

There was no information regarding blinding for outcome assessment in any of the studies (*Figure 2*). In the Sieira model, a history SCD was included as one of the predictors despite SCD being part of the outcome.^9^ In the Kawazoe model, the outcome of interest during follow-up was appropriate shocks/VF recordings on ICD, although 44.1% of patients (*n* = 63) did not undergo ICD implantation.^27^

The median number of MAE events utilized for model development analyses was 26 (range 7–114), corresponding to an events per candidate variable (EPV) ratio of 3.0 (range: 0.5 to 7.1). According to the PROBAST classification for model development studies, the number of participants with the outcome relative to the number of candidate predictor parameters is considered appropriate if EPV is ≥10.^17^ A median of 47 events (range: 7–75) were included in external validation analyses.

The most common model development approaches were Cox regression (*n* = 8, 80.0%) and logistic regression (*n* = 2, 20.0%) (see Supplementary material online, *Table S6*). Eight (80.0%) derivation studies employed univariable candidate predictor screening prior to multivariable modelling, whereas predictors for the PAT score were selected based on a systematic review of the literature.^11^ Regression coefficients or predictor weights were included in the final model in six cases. c-Statistic was reported in six (60.0%) model development analyses, of which five were adjusted via bootstrapping or cross-validation, and in all external validation studies as an estimate of model discrimination (see Supplementary material online, *Table S6*). Calibration measures were reported in four (26.7%) studies.^8,11,20,27^ In no case was information on the amount and handling of missing data provided.

Overall, all model development and external validation analyses were rated at high risk of bias in the PROBAST analysis domain. This was mainly due to a high risk of overfitting caused by low sample size relative to number of candidate predictors coupled with data-driven predictor selection methods, lack of correction for optimism in performance measures, or no assessment of model calibration (*Figure 2* and Supplementary material online, *Figure S1*).

### Model performance

**Figure 3:**
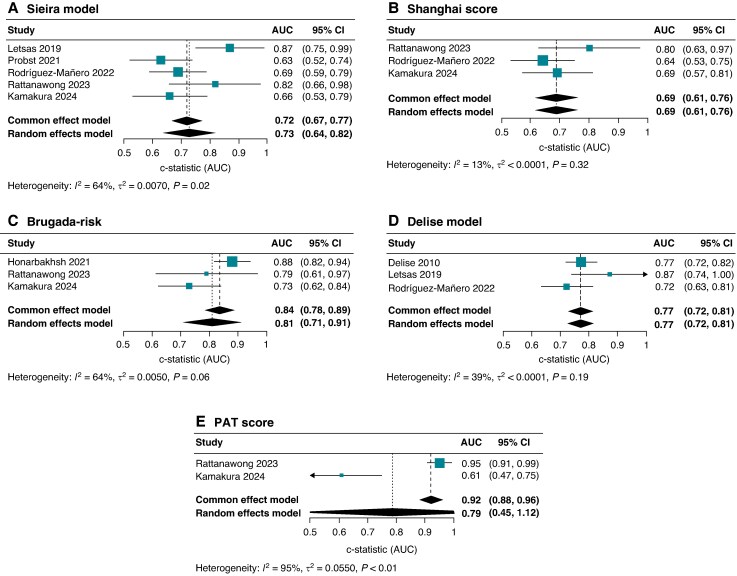
Forest plots with pooled c-statistics from individual multivariable models in patients without previous VT/VF or aborted SCD: (*A*) Sieira model, (*B*) Shanghai score, (*C*) BRUGADA-RISK, (*D*) Delise model, and (*E*) PAT score. SCD, sudden cardiac death; VF, ventricular fibrillation; VT, ventricular tachycardia.

Of the 11 unique models, c-statistics were reported in at least one independent external validation cohort in 72.7%.^9–11,24,25,27,28^ Pooled c-statistics for each multivariable score MAE prediction among primary prevention BrS patients across the derivation and/or external validation cohorts is presented in *Figure 3*. Pooled c-statistics in this group of patients showed good performance for Brugada-Risk (AUC 0.81, 95% CI 0.71–0.91; *I*^2^ 64%; three studies), fair for PAT (AUC 0.79, 95% CI 0.45–1.12; *I*^2^ 95%; two studies), Delise (AUC 0.77, 95% CI 0.72–0.81, *I*^2^ 39%, three studies), and Sieira (AUC 0.73, 95% CI 0.64–0.82; *I*^2^ 64%; five studies), and moderate for Shanghai (AUC 0.69, 95% CI 0.61–0,76; *I*^2^ 13%; three studies). It was not possible to pool data for the Subramanian, Letsas, Okamura, and Kawazoe models as c-statistics were only reported by one study.^22,24,25,27^ Additionally, for Shinohara *et al.*^23^ and Delinière *et al.*,^30^ data on model discrimination was not reported. Individual c-statistics among all derivation/validation cohorts are detailed in Supplementary material online, *Table S6*.

Letsas *et al.*^25^ externally validated and reported the negative predictive value (NPV) of three different models in patients without a history of cardiac arrest. They found an overall excellent NPV of 96% for the Delise model, 100% for the Sieira score, and 100% for the Okamura model. A PAT score of ≥10 had a very high NPV, 99.5%, among primary prevention BrS patients.^11^ For those with a Brugada-risk score of 0, freedom from events at 5 years was 98.5%, whilst for patients with 1 risk variable, this ranged between 94.1% and 96.4%.^8^

**Figure 4:**
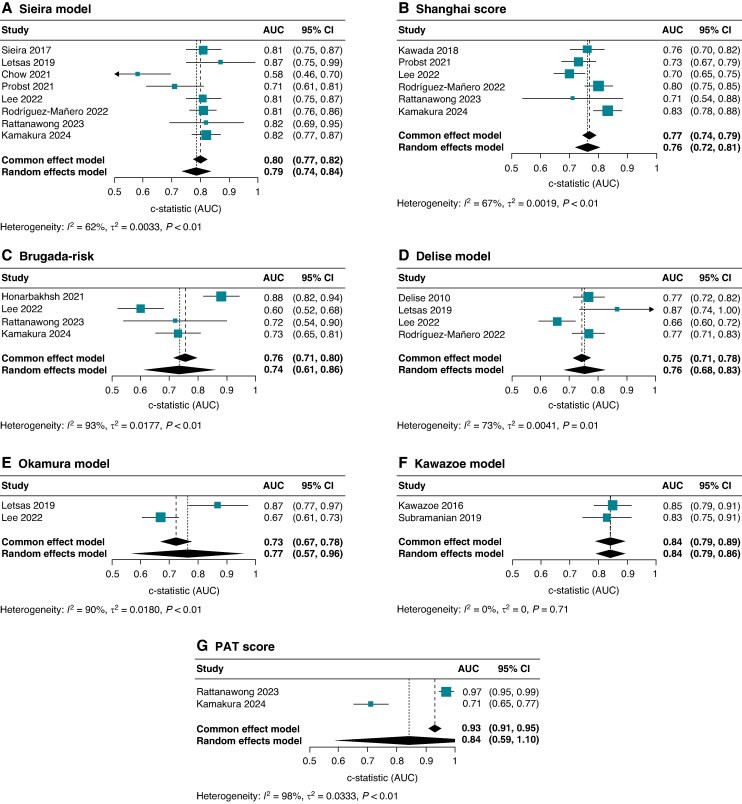
Forest plots with pooled c-statistics from individual multivariable models: (*A*) Sieira model, (*B*) Shanghai score, (*C*) BRUGADA-RISK, (*D*) Delise model, (*E*) Okamura model, (*F*) Kawazoe model, and (*G*) PAT score.

When including all available data (i.e. primary and secondary prevention BrS patients), the overall discriminative power improved for PAT (AUC 0.84, 95% CI 0.59–1.10, *I*^2^ 98%; two studies), Sieira (AUC 0.79, 95% CI 0.74–0.84, *I*^2^ 62%; eight studies), and Shanghai (AUC 0.76, 95% CI 0.72–0.81, *I*^2^ 67%; six studies) (*Figure 4*). In this setting, Sieira *et al.*^9^ reported event-free survival rates at 10 years of 97.2% and 96.4% for patients with scores of 0 and 1, respectively.

## Discussion

In this systematic review, we have performed a critical appraisal and pooled analysis of multiparametric clinical scores to predict MAE in BrS. The main findings were as follows:

(i) A total of 11 unique multivariable risk scores were identified, of which 8 (72.7%) were externally validated by independent groups.
(ii) All the development and/or external validation model analyses were assessed as having an overall high risk of bias, mainly due to inappropriate inclusion/exclusion criteria, predictor selection, low number of events per variable (median 3 EPV), and underreporting of model performance measures.
(iii) Pooled c-statistics for each unique model show an overall high heterogeneity and lower discriminative power than originally reported.
(iv) When applied to patients without previous MAE, Brugada-Risk had the best discriminative power (pooled AUC 0.81).
(v) The performance of Sieira, PAT, and Shanghai models improves in cohorts including primary and secondary prevention BrS patients.

At present, almost two thirds of patients with BrS are asymptomatic at the time of the diagnosis, and up to 0.2–0.6%/year will eventually develop ventricular arrhythmia or SCD as the initial form of presentation.^4,32,33^ General preventive measures, including aggressive treatment of fever, avoiding dehydration and drugs that may induce ST-segment elevation in right precordial leads (Class I anti-arrhythmics, some anaesthetics, and psychotropic drugs), and avoiding recreational substances such as cocaine, cannabis and of excessive alcohol intake are recommended for all patients, as these can exacerbate type 1 pattern and trigger VF.^3^ A review of cases of 74 cases of ‘drug-induced Brugada syndrome’ from non-cardiac medications, in which 23 individuals experienced spontaneous VF, was predominantly composed of young adult males, with drug toxicity appearing to play an important role.^34^

However, identifying asymptomatic BrS patients who will develop MAE remains a challenge of current day cardiac electrophysiology and requires individualized patient assessment.^3,7^ Prior studies have examined the value of routine clinical, electrocardiogram (ECG), EPS, and genetic variables in stratifying arrhythmic risk finding that a combined multiparametric approach may be more effective in predicting outcomes.^2,5,6,13,35^ This is of utmost importance, as the decision for ICD implantation must balance the benefits of preventing sudden death in at-risk patients against the rate of inappropriate shocks and long-term device-related complications (including lead-related issues and mortality), which can reach as high as 4–6% per year in young populations with inherited arrhythmia syndromes.^36–38^ Implantable cardiac monitors have been suggested for BrS with unexplained syncope (Class of recommendation IIa, level of evidence C), and utilized for patients where uncertainty exists in the indication for an ICD.^3,39,40^

### Quality of the assessed models

As a results of increasing publications on prediction models, health care providers frequently struggle to determine which to use, for whom or in which contexts.^17^ Against this background, the PROBAST tool was developed to specifically examine the risk of bias and applicability of a given diagnostic or prognostic score to the intended population and setting.^17^ Because PROBAST is not validated for the assessment of AI-derived models, in the work Lee *et al.*,^26^ only the external validation of existing risk scores was considered.

The major methodological limitations across studies were the use of inappropriate inclusion or exclusion patient criteria, selection of predictors, low number of events, and lack of appropriate internal and external validation of both discrimination and calibration. Specifically, all studies were judged to have an overall high risk of bias, with the majority (56%) also having major concerns regarding applicability. Almost one third of model development analyses and all external validation cohorts included patients with past history of ventricular arrhythmia or cardiac arrest, which may significantly affect the results. This may be particularly misleading, as good performance in external validation does not guarantee generalizability, especially when the population does not align with the intended use or clinical context.^41^

Bias on the outcome and analysis domains was deemed unclear or high for all studies due to small population size and low MAE per candidate predictor ratio, far less than the recommended minimum of 10–20 to reduce the probability of model overfitting.^17,42^ Among model development analyses, 40% did not report c-statistic values and 70% lacked any appropriate calibration assessment.^43^ Additionally, no information was provided regarding blinding for predictor and outcome assessment, nor was there a description of the amount and/or management of missing data.

### Model performance

Readily available variables during routine management of BrS patients, such as clinical history (history of syncope and family history of SCD) and ECG findings (spontaneous type 1 Brugada pattern, early repolarization pattern in inferolateral/peripheral leads and T-peak to T-end duration ≥ 100 ms), were among the most frequently included predictors in the final risk models.

Taken together, pooled c-statistics for each unique model was rather reasonable to moderate (AUC ranging from 0.74 to 0.84), and high heterogeneity was present in most cases. Interestingly, summary estimates were overall more modest when compared to the ones reported in the original publications. Significant variations in c-statistics and heterogeneity reflect differences in both the baseline characteristics of the population and absolute number of events, hindering the widespread applicability of the models. In this regard, ethnicity, family history, and genetics may play a pivotal role. Compared to white patients, Asians exhibit a higher prevalence of BrS and seemingly a greater incidence of SCD.^44^ Further research is required on the performance of these risk stratification models across different ethnicities, and how it compares to the prediction based on SCN5A status^45^ or obtained via polygenic risk scores.^46,47^ While genetic risk stratification is still evolving and requires further evaluation, SCN5A loss-of-function mutations, along with single nucleotide polymorphisms (SNPs) at other loci identified in genome-wide association studies (GWAS), have been variably associated with worse outcomes.^45–47^ Interestingly, a recent study has proposed a clinical arrhythmic prediction score for patients with an SCN5A loss-of-function variant.^48^

When utilized in primary prevention BrS patients, most models performed worse than initially reported in the primary publications. Nonetheless, they have been found to maintain an excellent NPV when all risk variables are absent (ranging from 96% to 100%). While prediction models may be less helpful than previously though in selecting patients for prophylactic ICD, withholding implantation in those without any of the identified high-risk criteria may still be appropriate.

### Clinical implications

To the best of our knowledge, this is the first study to systematically assess the quality of the multivariable risk scores predicting MAE in BrS patients. Using a dedicated tool for risk of bias assessment, we have demonstrated that there is significant room for improvement in the methodology of model development, validation, and reporting. Against this background, summary estimates for each predictive model were also provided, for all comers BrS patients, as well as for those in primary prevention.

Given the caveats in the available evidence, we propose the following key requirements to standardize the reporting and enhance the methodological robustness of future scores: (i) selection of derivation and validation cohorts from multicentre prospective registries using predefined inclusion and exclusion criteria that align with the population for which the model is intended; (ii) consistent syndrome definition (both temporal and geographical); (iii) sample size calculations ensuring an adequate ratio of EPV (>20); (iv) consistent predictors and outcome definitions for all patients and appropriate reporting and handling of any missing data; (v) blinded predictor and outcome assessment; (vi) appropriate internal validation and reporting of the discriminative power (AUC and respective 95% CI) and calibration (e.g. calibration plot); and (vii) external validation in an independent cohort. The added value of a prediction model for selecting BrS patients for prophylactic ICD should ideally be tested against current standard of care in a dedicated clinical outcomes randomized trial. We believe that these recommendations can be extended to different settings and not limited to BrS. In fact, risk models for other inherited cardiac arrhythmic syndromes and genetic cardiomyopathies may also exhibit comparable deficiencies due to the significant challenges associated with their development in the context of evolving definitions, low incidence, and low event rates. A similar methodological appraisal of these risk scores, along with adherence to standardized development criteria, would be valuable for better informing future decision-making and recommendations.

Recent advances in the understanding of risk stratification and genetic testing in inherited arrhythmic syndromes are expected to enhance the selection of tailored therapies in the near future. The presence of rare SCN5A variants may associate with worse arrhythmic outcomes. It is increasingly recognized that loss-of-function variants in SCN5A are linked to lethal events, highlighting their potential role in improving risk stratification.^49^ In the absence of these variants, BrS is considered to be largely polygenic, and with the advent of GWAS, there has been increased focus on SNPs as potential disease modifiers.^50^ While individual SNPs typically exhibit very small effect sizes in the occurrence of a given phenotype, when combined into polygenic scores, they may provide valuable insights into disease pathophysiology and severity.^35,50^ Additional advances in our understanding of the genotype–phenotype correlation will be essential for further refining risk stratification, identifying new therapeutic targets, and introducing gene-specific therapies, which are becoming increasingly available for familial arrhythmogenic conditions.^47,50,51^ The integration of clinical and genetic information will likely soon become the new standard for risk stratification.

### Limitations

Although this systematic review provides a comprehensive analysis of existing BrS MAE prediction scores, the results should be interpreted considering some limitations. Firstly, we excluded studies in which models were developed using AI. This was due to lack of traceability of the included parameters, and because the PROBAST tool was not validated in this context. A novel risk of bias instrument, the PROBAST-AI, to specifically address these studies, is in its early development.^52^ This review did not include risk scores based on genomics, which are currently limited to the research setting as our aim was to assess models containing clinical predictors routinely collected as part of standard care. Thirdly, most of the study cohorts included patients spanning more than two decades, in which the definition of BrS, scientific knowledge on risk predictors, and emerging risk factors such as positive genetic testing have changed dramatically. Incorporation of type-1 Brugada pattern electrocardiograms from second and third intercostal spaces or following exposure to sodium channel-blocker drugs are diagnostic features that were not present in earlier cohorts and account for some of the observed heterogeneity. Finally, while we report pooled AUC for individual models in the primary prevention setting, the limited number of external validation studies and participants warrants cautious interpretation before drawing definitive conclusions. For example, the Brugada-Risk score had excellent discrimination in its original derivation cohort, with an AUC of 0.88, but a more modest performance in subsequent validation cohorts, with AUC of 0.79 and 0.73.

## Conclusions

This systematic review highlights significant shortcomings in the development and validation of previous MAE prediction models in BrS patients. All models were found to carry a high risk of bias, primarily due to issues in model development and inadequate evaluation, which translates in high heterogeneity. Model performance in the primary prevention setting, and following external validation, was modest to fair for most models. Only one of the assessed scores, Brugada-Risk, had good discrimination when validated externally in primary prevention BrS patients.

Our findings provide valuable insights for improving the methodology of future research, with the goal of developing a reliable clinical tool to identify patients who may benefit from prophylactic ICD implantation.

## Supplementary Material

euaf091_Supplementary_Data

## Data Availability

The data supporting the findings of this study are available within the article and its Supplementary material online.
