# Supplementary material for: Multiparametric models for predicting major arrhythmic events in Brugada syndrome: a systematic review and critical appraisal
Source: Europace. 2025 May 2;27(5):euaf091. doi: 10.1093/europace/euaf091 (PMC12092914; doi:10.1093/europace/euaf091)

**SUPPLEMENTARY MATERIAL**

**Supplementary Table 1** – Complete search strategy.

| **Ovid MEDLINE(R) ALL <1946 to September 16, 2024>** |
| --- |
| 1 Brugada Syndrome/ (3994) |
| 2 brugada*.mp. (6287) |
| 3 1 or 2 (6287) |
| 4 exp Risk/ (1432923) |
| 5 (risk* adj4 (stratif* or model* or assessment* or predict* or factor* or characteristic*)).mp. (1861518) |
| 6 (clinical characteristic* or multiparametric or score*).mp. (1522637) |
| 7 4 or 5 or 6 (3314163) |
| 8 exp Death, Sudden/ (39363) |
| 9 Arrhythmias, Cardiac/ (68826) |
| 10 Ventricular Fibrillation/ (18101) |
| 11 Tachycardia, Ventricular/ (17389) |
| 12 (death or arrhythm* or arrhythmi* or ventric* tachycardia or ventric* fibrillation).mp. (1209686) |
| 13 8 or 9 or 10 or 11 or 12 (1211689) |
| 14 3 and 7 and 13 (1084) |
|  |
| **Embase <1974 to 2024 September 16>** |
| 1 Brugada syndrome/ (8603) |
| 2 brugada*.tw. (7217) |
| 3 1 or 2 (9639) |
| 4 risk model/ (4066) |
| 5 exp risk/ (3312377) |
| 6 prediction/ (556487) |
| 7 scoring system/ (334274) |
| 8 (risk* adj4 (stratif* or model* or assessment* or predict* or factor* or characteristic*)).tw. (1624592) |
| 9 (clinical characteristic* or multiparametric or score*).tw. (2328223) |
| 10 4 or 5 or 6 or 7 or 8 or 9 (6036996) |
| 11 exp sudden cardiac death/ (26515) |
| 12 heart ventricle arrhythmia/ (36970) |
| 13 heart ventricle fibrillation/ (38532) |
| 14 heart ventricle tachycardia/ (55063) |
| 15 (death or arrhythmi* or ventric* tachycardia or ventric* fibrillation).tw. (1501358) |
| 16 11 or 12 or 13 or 14 or 15 (1537935) |
| 17 3 and 10 and 16 (2542) |
|  |
| **Cochrane Library** |
| #1 MeSH descriptor: [Brugada Syndrome] this term only (32) |
| #2 brugada*:ti,ab,kw (79) |
| #3 #1 or #2 (79) |
| #4 MeSH descriptor: [Risk] explode all trees (56723) |
| #5 (risk* NEAR (stratif* or model* or assessment* or predict* or factor* or characteristic*)):ti,ab,kw (129300) |
| #6 (clinical NEXT characteristic* or multiparametric or score*):ti,ab,kw (380171) |
| #7 #4 or #5 or #6 (490343) |
| #8 MeSH descriptor: [Death, Sudden] explode all trees (1234) |
| #9 MeSH descriptor: [Arrhythmias, Cardiac] this term only (2530) |
| #10 MeSH descriptor: [Ventricular Fibrillation] this term only (697) |
| #11 MeSH descriptor: [Tachycardia, Ventricular] this term only (951) |
| #12 (death or arrhythm* or ventric* NEXT tachycardia or ventric* NEXT fibrillation):ti,ab,kw (98389) |
| #13 #8 or #9 or #10 or #11 or #12 (98468) |
| #14 #3 and #7 and #13 (25) |

**Supplementary Table 2** – Data extraction form and PROBAST assessment of risk of bias.

| **Data field** | **Answer options and instructions** |
| --- | --- |
| Author (publication year) |  |
| Model name |  |
| Type of prediction model evaluation | -Development only -Development and internal validation -Development and temporal validation -Development (+/- internal validation) and external validation  -External validation only |
| Notes: Type of prediction model evaluation |  |
| Specify countries |  |
| Study years |  |
| Study design | -Prospective cohort Retrospective cohort -Nested case-control -Nonnested case-control -Case-cohort -Randomized trial participants -Registry data -Other -Unclear |
| Notes: Study design |  |
| Recruitment method | -Consecutive -Other -Unclear |
| Notes: Recruitment method |  |
| Number of centers from where patients were recruited | -Monocenter -Multicenter -Statewide -Nationwide -Not reported |
| Age | Specify median (IQR) or mean (SD) (round to 1 decimal) |
| Male sex | N (%) |
| Prior syncope | N (%) |
| Family history of SCD | N (%) |
| Spontaneous Brugada type 1 pattern | N (%) |
| Positive EPS study | N (%) |
| Positive genetic testing | N (%) |
| Previous aborted SCD or VF | N (%) |
| PROBAST signaling question 1.1: Were appropriate data sources used, e.g., cohort, RCT, or nested case-control study data? | -Yes/probably yes: If a cohort design (including RCT or proper registry data) or a nested case-control or case-cohort design (with proper adjustment of the baseline risk/hazard in the analysis) has been used. -No/probably no: If a nonnested case-control design has been used. -No information: If the method of participant sampling is unclear. |
| Notes: PROBAST signaling question 1.1 |  |
| PROBAST signaling question 1.2: Were all inclusions and exclusions of participants appropriate? | -Yes/probably yes: If inclusion and exclusion of participants was appropriate, so participants correspond to unselected participants of interest. -No/probably no: If participants are included who would already have been identified as having the outcome and so are no longer participants at risk of developing outcome, or if specific subgroups are excluded that may have altered the performance of the prediction model for the intended target population. -No information: When there is no information on whether inappropriate inclusions or exclusions took place. |
| Notes: PROBAST signaling question 1.2 |  |
| PROBAST risk of bias introduced by participants or data sources | -Low risk of bias: If the answer to all signaling questions is "Yes" or "Probably yes", then risk of bias can be considered low. If ≥1 of the answers is "No" or "Probably no", the judgment could still be "Low risk of bias", but specific reasons should be provided why the risk of bias can be considered low. -High risk of bias: If the answer to any of the signaling questions is "No" or "Probably no", there is a potential for bias, except if defined at low risk of bias above. - Unclear risk of bias: If relevant information is missing for some of the signaling questions and non of the signaling questions is judged to put this domain at high risk of bias. |
| Rationale for PROBAST participants domain risk of bias rating |  |
| Can the number of candidate predictors be determined? | - Yes - No, but a minimum can be estimated - No |
| Number of candidate predictors (total or minimum) | This includes all predictors, also those considered at the univariable analysis stage. Specificy the number of candidate predictors. |
| Additional degrees of freedom candidate predictors (total or minimum) |  |
| Total degrees of freedom candidate predictors (total or minimum) |  |
| PROBAST signaling question 2.1: Were predictors defined and assessed in a similar way for all participants? | -Yes/probably yes: If definitions of predictors and their assessment were similar for all participants. -No/probably no: If different definitions were used for the same predictor or if predictors requiring subjective interpretation were assessed by differently experienced assessors. -No information: If there is no information on how predictors were defined or assessed. |
| Notes: PROBAST signaling question 2.1 |  |
| PROBAST signaling question 2.2: Were predictor assessments made without knowledge of outcome data? | -Yes/probably yes: If outcome information was stated as not used during predictor assessment or was clearly not (yet) available to those assessing predictors. -No/probably no: If it is clear that outcome information was used when assessing predictors. -No information: No information on whether predictors were assessed without knowledge of outcome information |
| Notes: PROBAST signaling question 2.2 |  |
| PROBAST signaling question 2.3: Are all predictors available at the time the model is intended to be used? | -Yes/probably yes: All included predictors would be available at the time the model is intended to be used for prediction. -No/probably no: Predictors would not be available at the time the model is intended to be used for prediction. -No information: No information on whether predictors would be available at the time the model is intended to be used for prediction. |
| Notes: PROBAST signaling question 2.3 |  |
| PROBAST risk of bias introduced by predictors or their assessment | -Low risk: If the answer to all signaling question is "Yes" or "Probably yes", then risk can be considered low. If ≥1 of the answers is "No" or "Probably no", the judgment could still be "Low risk" but specific reasons should be provided why the risk can be considered low, e.g., use of objective predictors not requiring subjective interpretation. -High risk: If the answer to any of the signaling questions is "No" or "Probably no", there is a potential for bias. -Unclear risk: If relevant information is missing for some of the signaling questions and none of the signaling questions is judged to put the domain at high risk. |
| Rationale for PROBAST predictors domain risk of bias rating |  |
| Outcome definition |  |
| Method for outcome determination |  |
| Follow-up time |  |
| PROBAST signaling question 3.1: Was the outcome determined appropriately? | -Yes/probably yes: If a method of outcome determination has been used which is considered optimal or acceptable. Note: this is about level of measurement error within the method of determining the outcome (see concerns for applicability about whether the definition of the outcome method is appropriate). -No/probably no: If a clearly suboptimal method has been used that causes unacceptable error in determining outcome status in participants. -No information: No information on how outcome was determined. |
| Notes: PROBAST signaling question 3.1 |  |
| PROBAST signaling question 3.2: Was a prespecified or standard outcome definition used? | -Yes/probably yes: If the method of outcome determination is objective, or if a standard outcome definition is used, or if prespecified categeories are used to group outcomes. -No/probably no: If the outcome definition was not standard and not prespecified. -No information: No information on whether the outcome definition was prespecified or standard. |
| PROBAST signaling question 3.3: Were predictors excluded from the outcome definition? | -Yes/probably Yes: If none of the predictors are included in the outcome definition. -No/probably No: If ≥1 of the predictors forms part of the outcome definition. -No information: No information on whether predictors are excluded from the outcome definition. |
| PROBAST signaling question 3.4: Was the outcome defined and determined in a similar way for all participants? | -Yes/probably yes: If outcomes were defined and determined in a similar way for all participants. -No/probably no: If outcomes were clearly defined and determined in a different way for some participants. -No information: No information on whether the outcome defined and determined in a similar way for all participants |
| PROBAST signaling question 3.5: Was the outcome determined without knowledge of predictor information? | -Yes/probably yes: If predictor information was not known when determining the outcome status, or outcome status determination is clearly reported as determined without knowledge of predictor information. -No/probably no: If it is clear that predictor information was used when determining the outcome status. -No information: No information on whether outcome was determined without knowledge of predictor information. |
| Notes: PROBAST signaling question 3.5 |  |
| PROBAST signaling question 3.6: Was the time interval between predictor assessment and outcome determination appropriate? | -Yes/probably yes: If the time interval between predictor assessment and outcome determination was appropriate to enable the correct type and representative number of relevant outcomes to be recorded, or if no information on the time interval is required to allow a representative number of the relevant outcome occur or if predictor assessment and outcome determination were from information taken within an appropriate time interval. -No/probably no: If the time interval between predictor assessment and outcome determination is too short or too long to enable the correct type and representative number of relevant outcomes to be recorded. -No information: If no information was provided on the time interval between predictor assessment and outcome determination |
| Notes: PROBAST signaling question 3.6 |  |
| PROBAST risk of bias introduced by the outcome or its determination | -Low risk of bias: If the answer to all signaling questions is "Yes" or "Probably yes", then risk of bias can be considered low.If ≥1 of the answers is "No" or "Probably no" , the judgement could still be low risk of bias, but specific reasons should be provided why the risk of bias can be considered low, e.g., when the outcome was determined with knowledge of predictor information but the outcome assessment did not require much interpretation by the assessor (e.g., death regardless of cause). -High risk of bias: If the answer to any of the signaling questions is "No" or "Probably no", there is a potential for bias. -Unclear risk of bias: If relevant information about the outcome is missing for some of the signaling questions and none of the signaling questions is judged to put this domain at high risk of bias. |
| Rationale for PROBAST outcome domain risk of bias rating | Provide specific reasons for classification of bias if necessary |
| Total number of participants for model development | Specify number (exclude participants with missing data if a complete case analysis was done and exclude participants used for testing) |
| Number of participants with outcome for model development | Specify number (exclude participants with missing data if a complete case analysis was done and exclude participants used for testing) |
| Number of events per candidate predictor (EPV) for model development | See Predictors section for candidate predictor count |
| Handling of missing data | -Complete-case analysis -Single imputation -Multiple imputation -Sensitivity analysis -Variable omission -Indicator methods -Other -Not reported |
| Notes: Handling of missing data |  |
| Modelling method | -Logistic regression -Multinomial/polytomous regression -Cox proportional hazards regression -Accelerated failure time analysis (e.g. Weibull model) -Other parametric survival model -Support vector machine -Neural net -Tree-based -Other -Not reported -Unclear |
| Notes: Modelling method |  |
| Method for selection of predictors in multivariable model (this refers to selection prior to data-driven multivariable selection such as backwards or Lasso) | -All candidate predictors -Pre-selection based on univariable analysis -Principal component analysis -Variance inflation factor / multicollinearity -Not applicable (medical imaging) -Other -Not reported -Unclear |
| Notes: Method for selection of predictors in multivariable model |  |
| Method for selection of predictors during multivariable analysis | -All predictors forced in model -BW (Stepwise) selection -FW (Stepwise) selection -Added value -Lasso -Not applicable -Unclear -Not reported -Other |
| Notes: Method for selection of predictors during multivariable analysis |  |
| Apparent performance: Calibration plot presented? | -Yes -No -No, but possible to construct based on provided information -Not possible |
| Apparent performance: C-statistic point estimate | C-statistic, specify value as 0.xx |
| Apparent performance: C-statistic 95% CI | C-statistic 95% confidence interval (0.xx to 0.xx) |
| Apparent performance: Sensitivity point estimate | Sensitivity (%), specify value as number between 0 and 100, round to percentage point |
| Apparent performance: Sensitivity 95% CI | Sensitivity (95% confidence interval xx to xx) |
| Apparent performance: Specificity point estimate | Specificity (%), specify value as number between 0 and 100, round to percentage point |
| Apparent performance: Specificity 95% CI | Specificity (95% confidence interval xx to xx) |
| Apparent performance: Hosmer-Lemeshow test result presented | -Yes -No |
| Apparent performance: Decision curve / net benefit analysis presented | -Yes -No |
| Apparent performance: Other measures present | List any other performance measures |
| Notes: Apparent performance |  |
| Technique used for internal validation | -(Random) split of data set into development and testing datasets -Temporal split of data set into development and testing set -Internal validation by resampling of same data set (e.g. bootstrap (specify the number of samples) -Cross-validation (x-fold, specifiy in note column) -Not applicable -Other |
| Notes: Technique used for internal validation |  |
| Shrinkage of predictor weights or regression coefficients | -Not performed -Heuristic shrinkage (uniform) -Calibration slope assessed with bootstrapping (uniform) -Penalized maximum likelihood estimation -Lasso/Ridge -Not directly applicable -Other -Unclear |
| Total number of participants for model validation in case of train-test split (e.g. random or temporal) | Specify number (exclude participants with missing data if a complete case analysis was done) |
| Number of participants for model validation with AFACS in case of train-test split (e.g. random or temporal) | Specify number |
| Internal/temporal validation performance: Calibration plot presented? | -Yes -No -No but possible to construct based on provided information -Not possible |
| Internal/temporal validation performance: C-statistic point estimate | C-statistic, specify value as 0.xx |
| Internal/temporal validation performance: C-statistic 95% CI | C-statistic 95% confidence interval (0.xx to 0.xx) |
| Internal/temporal validation performance: Sensitivity point estimate | Sensitivity (%), specify value as number between 0 and 100, round to percentage point |
| Internal/temporal validation performance: Sensitivity 95% CI | Sensitivity (95% confidence interval xx to xx) |
| Internal/temporal validation performance: Specificity point estimate | Specificity (%), specify value as number between 0 and 100, round to percentage point |
| Internal/temporal validation performance: Specificity 95% CI | Specificity (95% confidence interval xx to xx) |
| Internal/temporal validation: perform ce: Hosmer-Lemeshow test result presented | -Yes -No |
| Internal/temporal validation performance: Decision curve / net benefit analysis presented | -Yes -No |
| Internal/temporal validation performance: Other measures presented | List any other performance measures |
| Notes: Internal/temporal validation performance |  |
| Did model being externally validated provide a way to estimate predicted probability in the development paper? | -Yes, full regression formula -Yes, nomogram -Yes, online tool -No |
| How was model assessed in external validation? | -As predicted probability -Sum of integer scoring system -Both |
| Total number of participants for model validation in case of external validation | Specify number |
| Total number of participants for model validation with AFACS in case of external validation | Specify number (exclude participants with missing data if a complete case analysis was done) |
| How does the external validation dataset differ from the model development dataset? | -Geographic (new centers, different country) -Geographic (new centers, same country) -Different outcome -Different patient population |
| External validation performance: Calibration plot presented? | -Yes -No -No but possible to construct based on provided information -Not possible |
| External validation performance: C-statistic point estimate | C-statistic, specify value as 0.xx |
| External validation performance: C-statistic 95% CI | C-statistic 95% confidence interval (0.xx to 0.xx) |
| External validation performance: Sensitivity point estimate | Sensitivity (%), specify value as number between 0 and 100, round to percentage point |
| External validation performance: Sensitivity 95% CI | Sensitivity (95% confidence interval xx to xx) |
| External validation performance: Specificity point estimate | Specificity (%), specify value as number between 0 and 100, round to percentage point |
| External validation performance: Specificity 95% CI | Specificity (95% confidence interval xx to xx) |
| External validation: perform ce: Hosmer-Lemeshow test result presented | -Yes -No |
| External validation performance: Decision curve / net benefit analysis presented | -Yes -No |
| External validation performance: Other measures presented | List any other performance measures |
| Notes: External validation performance |  |
| Final model presented including intercept and predictor weights? | -Yes -No |
| Notes: Final model presented including intercept and predictor weights? |  |
| Final model presented as nomogram? | -Yes -No -Unclear |
| Notes: Final model presented as nomogram? |  |
| Final model presented as online tool? | -Yes -No |
| Notes: Final model presented as online tool? |  |
| Final model presented as a chart/diagram (besides nomogram)? | -Yes -No |
| Notes: Final model presented as a chart/diagram (besides nomogram)? |  |
| Final model presented as partial formula (i.e., hazard/odd ratio's, no intercept/baseline hazard? | -Yes -No |
| Notes: Final model presented as partial formula? |  |
| Final model presented as integer sum score / risk index? | -Yes -No |
| Notes: Final model presented as integer sum score / risk index? |  |
| PROBAST signaling question 4.1: Were there a reasonable number of participants with the outcome? | -Yes/probably yes: For model development studies, if the number of participants with the outcome relative to the number of candidate predictors parameters is ≥20 (EPV ≥20). For EPV between 10 and 20, the item should be rated as either probably yes or probably no, depending on the outcome frequency, overall model performance, and distribution of the predictors in the model. For model validation studies, if the number of participants with the outcome is ≥100. -No/probably no: For model development studies, if the number of participants with the outcome relative to the number of candidate predictor parameters is <10 (EPV <10). For model validation studies, if the number of participants with the outcome is <100. -No information: For model development studies, no information on the number of candidate predictor parameters or number of participants with the outcome, such that the EPV cannot be calculated. For model validation studies, no information on the number of participants with the outcome. |
| Notes: PROBAST signaling question 4.1 |  |
| PROBAST signaling question 4.2: Were continuous and categorical predictors handled appropriately? | -Yes/probably yes: If continuous predictors are not converted into ≥2 categories when included in the model (i.e., dichotomized or categorized), or if continous predictors are examined for nonlinearity using, for example, fractional polynomials or restricted cubic splines, or if categorical predictor groups are defined using a prespcified method. For model validation studies, if continious predictors are included using the same definitions or transformations, and categorical variables are categorized using the same cut points, as compared with the development study. -No/probably no: If categorical predictor group definitions do not use a prespecified method. For model development studies, if continous predictors are converted into ≥2 categories when included in the model. For model validation studies, if continuous predictors are included using different definitions or transformations, or categorical variables are categorized using different cut points, as compared with the development study. -No information: No information on whether continuous predictors are examined for nonlinearity and no information on how categorical predictor groups are defined. For model validation studies, no information on whether the same definitions or transformations and the same cut points are used, as compared with the development study. |
| Notes: PROBAST signaling question 4.2 |  |
| PROBAST signaling question 4.3: Were all enrolled participants included in the analysis? | -Yes/probably yes: If all participants enrolled in the study are included in the data analysis. -No/probably no: If some or a subgroup of participants are inappropriately excluded from the analysis. -No information: No information on whether all enrolled participants are included in the analysis. |
| Notes: PROBAST signaling question 4.3 |  |
| PROBAST signaling question 4.4: Were participants with missing data handled appropriately? | -Yes/probably yes:If there are no missing values of predictors or outcomes and the study explicitly reports that participants are not excluded on the basis of missing data, or if missing values are handled using multiple imputation. -No/probably no: If participants with missing data are omitted from the analysis, or if the method of handling missing data is clearly flawed, e.g., missing indicator method or inappropriate use of last value carried forward, or if the study had nog explicit mention of methods to handle missing data. -No information: If there is insufficient information to determine if the method of handling missing data is appropriate. |
| Notes: PROBAST signaling question 4.4 |  |
| PROBAST signaling question 4.5: Was selection of predictors based on univariable analysis avoided? | -Yes/probably yes: If the predictors are not selected on the basis of univariable analysis prior to multivariable modeling. -No/probably no: If the predictors are selected on the basis of univariable analysis prior to multivariable modeling. -No information: If there is no information to indicate that univariable selection is avoided. |
| Notes: PROBAST signaling question 4.5 |  |
| PROBAST signaling question 4.6: Were complexities in the data (e.g., censoring, competing risks, sampling of control participants) accounted for appropriately? | -Yes/probably yes: If any complexities in the data are accounted for appropriately, or if it is clear that any potential data complexities have been identified appropriately as unimportant. -No/probably no: If complexities in the data that could affect model performance are ignored. -No information: No information is provided on whether complexities in the data are present or accounted for appropriately if present. |
| Notes: PROBAST signaling question 4.6 |  |
| PROBAST signaling question 4.7: Were relevant model performance measures evaluated appropriately? | -Yes/probably yes: If both calibration and discrimination are evaluated appropriately (including relevant measures tailored for models predicting survival outcomes). -No/probably no: If both calibration and discrimination are not evaluated, or if only goodness-of-fit tests, such as the Hosmer-Lemeshow test, are used to evaluate calibration, or if models predicting survival outcomes performance measures accounting for censoring are not used, or if classification measures (like sensitivity, specificity, or predictive values) were presented using predicted probability tresholds dervied from the data set at hand. -No information: Either calibration or discrimination are not reported, or no information is provided as to whether appropriate performance measures for survival outcomes are used (e.g., references to relevant literature or specific mention of methods, such as using Kaplan-Meier estimates), or no information on tresholds for estimating classification measures is given. |
| Notes: PROBAST signaling question 4.7 |  |
| PROBAST signaling question 4.8: Were model overfiting and optimism in model performance accounted for? | -Yes/probably yes: If internal validation techniques, such as bootstrapping and cross-validation including all model development procedures, have been used to account for any optimism in model fitting, and subsequent adjustment of the model performance estimates have been applied. -No/probably no: If no internal validation has been performed, or if the bootstrapping or cross-validation did not include all model development procedures including any variable selection; if 'data leakage' was an issue (test data not completely independent of development data); if one cross-validation was used for parameter tuning and internal validation -No information: No information is provided on whether internal validation techniques, including all model development procedures, have been applied. |
| Notes: PROBAST signaling question 4.8 |  |
| PROBAST signaling question 4.9: Do predictors and their assigned weights in the final model correspond to the results from the reported multivariable analysis? | -Yes/probably yes: If the predictors and regression coefficients in the final model correspond to reported results from multivariable analysis. -No/probably no: If the predictors and regression coefficients in the final model do not correspond to reported results from multivariable analysis -No information: If it is unclear whether the regression coefficients in the final model correspond to reported results from multivariable analysis. Where applicable, specify separately for the original model and risk index, separated by ";" |
| Notes: PROBAST signaling question 4.9 |  |
| PROBAST risk of bias introduced by the analysis | -Low risk of bias: If the answer to all signaling questions is "Yes" or "Probably yes", then risk of bias can be considered low. If ≥1 of the answers is "No" or "Probably no", the judgement could still be low risk of bias, but specific reasons should be rovided why the risk of bias can be considered low. -High risk of bias: If the answer to any of the signaling questions is "No" or "Probably no", there is a potential for bias. -Unclear risk of bias: If relevant information about the analysis is missing for some of the signaling questions but none of the signaling questions is judged to put the analysis at high risk of bias. |
| Rationale for PROBAST analysis domain risk of bias rating | Provide specific reasons for classification of bias if necessary |
| PROBAST overall risk of bias | -Low risk of bias: If all domains were reated low risk of bias.  -High risk of bias: If at least one domain is judged to be at high risk of bias. -Unclear risk of bia: If an unclear risk of bias was noted in at least one domain and it was low risk for all other domains. |
| Rationale for overall PROBAST risk of bias rating | Provide specific reasons for classification of bias if necessary |

**Supplementary Table 3** – Motives of exclusion of studies after full-text review.

| **Author and year of publication** | **Title** | **Journal** | **Motive for exclusion** |
| --- | --- | --- | --- |
| Yonezu et al. 2021 (1) | Role of fragmented QRS and Shanghai score system in recurrence of ventricular fibrillation in patients with early repolarization syndrome | Ann Noninvasive Electrocardiol | Validation of Shanghai score in Early Repolarization Syndrome |
| Wilde 2018 (2) | The Shanghai Score System in Brugada Syndrome: Using it Beyond a Diagnostic Score | JACC Clin Electrophysiol | Editorial |
| Wijeyeratne et al. 2020 (3) | SCN5A Mutation Type and a Genetic Risk Score Associate Variably With Brugada Syndrome Phenotype in SCN5A Families | Circ Genom Precis Med | No clinical multiparametric model derivation or validation |
| Wei et al. 2022 (4) | Performance of Multiparametric Models in Patients With Brugada Syndrome: A Systematic Review and Meta-Analysis | Front Cardiovasc Med | Meta-analysis |
| Nakamura et al. 2023 (5) | Prediction of the Presence of Ventricular Fibrillation From a Brugada Electrocardiogram Using Artificial Intelligence | Circ J | No clinical multiparametric model derivation or validation. |
| Corcia et al. 2017 (6) | A Clinical Score Model to Predict Lethal Events in Young Patients (≤19 Years) With the Brugada Syndrome | Am J Cardiol | Only included children (<18 years) |
| Casu et al. 2021 (7) | Predictors of inappropriate shock in Brugada syndrome patients with a subcutaneous implantable cardiac defibrillator | J Cardiovasc Electrophysiol | Not predicting major arrhythmic events |
| Chung et al. 2022 (8) | Predictive risk models for forecasting arrhythmic outcomes in Brugada syndrome: A focused review | J Electrocardiol | Review |
| Lee et al. 2020 (9) | Territory-wide cohort study of Brugada syndrome in Hong Kong: predictors of long-term outcomes using random survival forests and non-negative matrix factorisation | Open Heart | AI generated model |
| Tse et al. 2020 (10) | Incorporating Latent Variables Using Nonnegative Matrix Factorization Improves Risk Stratification in Brugada Syndrome | J Am Heart Assoc | AI generated model |
| Tse et al. 2021 (11) | Automated Electrocardiogram Analysis Identifies Novel Predictors of Ventricular Arrhythmias in Brugada Syndrome | Front Cardiovasc Med | No clinical multiparametric model derivation or validation. |
| Milman et al. 2019 (12) | Time-to-first appropriate shock in patients implanted prophylactically with an implantable cardioverter-defibrillator: data from the Survey on Arrhythmic Events in BRUgada Syndrome (SABRUS) | Europace | Other outcome/ clinical setting |
| Tokioka et al. 2014 (13) | Electrocardiographic Parameters and Fatal Arrhythmic Events in Patients With Brugada Syndrome: Combination of Depolarization and Repolarization Abnormalities | J Am Coll Cardiol | No clinical multiparametric model derivation or validation. |
| McEnteggart et al. 2019 (14) | Prediction and Prevention of Sudden Death in the Brugada Syndrome | Am J Cardiol | Review |
| Morita et al. 2003 (15) | Risk Stratification for Asymptomatic Patients With Brugada Syndrome - Prediction of Induction of Ventricular Fibrillation by Noninvasive Methods | Circ J | No clinical multiparametric model derivation or validation. |

References for Supplementary Table 2:

1. Yonezu K, Shinohara T, Sato H, Hirota K, Kondo H, Fukui A, et al. Role of fragmented QRS and Shanghai score system in recurrence of ventricular fibrillation in patients with early repolarization syndrome. Ann Noninvasive Electrocardiol. 2021 Nov;26(6):e12873.

2. Wilde AAM. The Shanghai Score System in Brugada Syndrome: Using it Beyond a Diagnostic Score. JACC Clin Electrophysiol. 2018 Jun;4(6):731-732.

3. Wijeyeratne YD, Tanck MW, Mizusawa Y, Batchvarov V, Barc J, Crotti L, et al. SCN5A Mutation Type and a Genetic Risk Score Associate Variably With Brugada Syndrome Phenotype in SCN5A Families. Circ Genom Precis Med. 2020 Dec;13(6):e002911.

4. Wei HT, Liu W, Ma YR, Chen S. Performance of Multiparametric Models in Patients With Brugada Syndrome: A Systematic Review and Meta-Analysis. Front Cardiovasc Med. 2022 Apr 14;9:859771.

5. Nakamura T, Aiba T, Shimizu W, Furukawa T, Sasano T. Prediction of the Presence of Ventricular Fibrillation From a Brugada Electrocardiogram Using Artificial Intelligence. Circ J. 2023 Jun 23;87(7):1007-1014.

6. Gonzalez Corcia MC, Sieira J, Pappaert G, de Asmundis C, Chierchia GB, Sarkozy A, et al. A Clinical Score Model to Predict Lethal Events in Young Patients (≤19 Years) With the Brugada Syndrome. Am J Cardiol. 2017 Sep 1;120(5):797-802.

7. Casu G, Silva E, Bisbal F, Viola G, Merella P, Lorenzoni G, et al. Predictors of inappropriate shock in Brugada syndrome patients with a subcutaneous implantable cardiac defibrillator. J Cardiovasc Electrophysiol. 2021 Jun;32(6):1704-1711.

8. Chung CT, Bazoukis G, Radford D, Coakley-Youngs E, Rajan R, Matusik PT, et al. Predictive risk models for forecasting arrhythmic outcomes in Brugada syndrome: A focused review. J Electrocardiol. 2022 May-Jun;72:28-34.

9. Lee S, Zhou J, Li KHC, Leung KSK, Lakhani I, Liu T, et al. Territory-wide cohort study of Brugada syndrome in Hong Kong: predictors of long-term outcomes using random survival forests and non-negative matrix factorisation. Open Heart. 2021 Feb;8(1):e001505.

10. Tse G, Zhou J, Lee S, Liu T, Bazoukis G, Mililis P, et al. Incorporating Latent Variables Using Nonnegative Matrix Factorization Improves Risk Stratification in Brugada Syndrome. J Am Heart Assoc. 2020 Nov 17;9(22):e012714.

11. Tse G, Lee S, Li A, Chang D, Li G, Zhou J, Liu T, Zhang Q. Automated Electrocardiogram Analysis Identifies Novel Predictors of Ventricular Arrhythmias in Brugada Syndrome. Front Cardiovasc Med. 2021 Jan 14;7:618254.

12. Milman A, Hochstadt A, Andorin A, Gourraud JB, Sacher F, Mabo P, et al. Time-to-first appropriate shock in patients implanted prophylactically with an implantable cardioverter-defibrillator: data from the Survey on Arrhythmic Events in BRUgada Syndrome (SABRUS). Europace. 2019 May 1;21(5):796-802.

13. Tokioka K, Kusano KF, Morita H, Miura D, Nishii N, Nagase S, et al. Electrocardiographic parameters and fatal arrhythmic events in patients with Brugada syndrome: combination of depolarization and repolarization abnormalities. J Am Coll Cardiol. 2014 May 27;63(20):2131-2138.

14. McEnteggart S, Estes NAM 3rd. Prediction and Prevention of Sudden Death in the Brugada Syndrome. Am J Cardiol. 2019 Dec 1;124(11):1797-1802.

15. Morita H, Takenaka-Morita S, Fukushima-Kusano K, Kobayashi M, Nagase S, Kakishita M, et al. Risk stratification for asymptomatic patients with Brugada syndrome. Circ J. 2003 Apr;67(4):312-6.

**Supplementary Table 4** – List of candidate and final model predictors from included studies.

| **Category** | **Predictor** |
| --- | --- |
| **Demographics** | Age* |
|  | Sex |
| **Past medical history** | Syncope* |
|  | Arrhythmic syncope/ nocturnal agonal respiration* |
|  | Unexplained syncope* |
|  | Aborted SCD or previous VT/ VF* |
|  | Sinus node disease* |
|  | Atrial fibrillation/ atrial flutter* |
|  | MAE during drug challenge testing* |
| **Family history** | First- or second-degree relative with definite BrS * |
|  | Family history of SCD* |
| **ECG pattern** | Spontaneous Brugada type 1 pattern* |
|  | Drug-induced Brugada type 1 pattern* |
|  | Type 1 pattern in peripheral leads* |
|  | Heart rate |
|  | r-J interval in lead V1* |
|  | T-peak T-end ≥ 100ms * |
|  | QRS duration in lead V2* |
|  | QRS duration in lead V6* |
|  | T-wave alternace |
|  | Early repolarization in inferolateral/ peripheral leads* |
|  | Early repolarization with a notching/ slurring pattern |
|  | S-wave in lead I |
|  | aVR sign* |
|  | S wave upslope ratio > 0.8* |
|  | QRS fragmentation* |
|  | PR interval ≥ 200ms* |
|  | QTc interval |
|  | Duration of low amplitude signal (LAS40) in signal averaged ECG |
| **Electrophysiological study** | Inducible VT/VF in PES* |
|  | Ventricular effective refractory period <200 ms |
|  | HV interval |
| **Genetic testing** | SCN5A mutation* |

* Included in at least one multivariable prediction model.

Abbreviations as in Table 1.

| PAT score | |  | BRUGADA-RISK score | |
| --- | --- | --- | --- | --- |
| Risk factor | Score |  | Risk factor | Score |
| T-peak T-end≥100ms | 5 |  | Syncope | 12 |
| Arrhytmic or unexplained syncope | 5 |  | Type-1 pattern in peripheral leads | 9 |
| VT/VF during drug challenge testing | 4 |  | Early repolarization in inferolateral leads | 12 |
| PR≥200ms | 4 |  | Spontaneous type-1 pattern | 14 |
| aVR sign | 3 |  |  |  |
| Type-1 pattern in peripheral leads | 3 |  | Shanghai score |  |
| Early repolarization in inferolateral leads | 3 |  | Risk factor | Score |
| Fragmented QRS | 3 |  | Spontaneous type-1 pattern | 3.5 |
|  |  |  | Fever-induced type-1 pattern | 3 |
| Delinère score |  |  | Drug-induced type-1 pattern | 2 |
| Risk factor | Score |  | Unexplained SCD/VF | 3 |
| Type-1 pattern in peripheral leads | 1 |  | Nocturnal agonal respiration | 2 |
| Early repolarization in inferolateral leads | 1 |  | Suspected arrhythmic syncope | 2 |
| T-peak T-end≥100ms | 1 |  | Unexplained syncope | 1 |
| Age | modifier |  | AF or AFL < 30 years old | 0.5 |
|  |  |  | Family history of BrS | 2 |
| Okamura score |  |  | Suspicious familial SCD | 1 |
| Risk factor | Score |  | Unexplained family history of SCD < 45 years | 0.5 |
| Spontaneous type-1 pattern | 1 |  | Probable pathogenic mutation | 0.5 |
| Syncope | 1 |  |  |  |
| Inducible PES | 1 |  | Sieira score |  |
|  |  |  | Risk factor | Score |
| Subramanian score |  |  | Spontaneous type-1 pattern | 1 |
| Risk factor | Score |  | Previous sudden cardiac arrest | 4 |
| Spontaneous type-1 pattern | 1 |  | Syncope | 2 |
| Fragmented QRS in inferior leads | 1 |  | Family history of SCD < 35 years old | 1 |
| T-peak T-end≥100ms | 1 |  | Inducible EPS | 2 |
| S wave upslope duration ratio ≥ 0.8 | 1 |  | Sinus node dysfunction | 3 |
|  |  |  |  |  |
| Shinohara score |  |  | Letsas score |  |
| Risk factor | Score |  | Risk factor | Score |
| Spontaneous type-1 pattern | 1 |  | Spontaneous type-1 pattern | 1 |
| Family history of SCD | 1 |  | Syncope | 1 |
| QRS duration in lead V2 >90ms | 1 |  | Family history of SCD | 1 |
| J wave in inferolateral leads | 1 |  | Fragmented QRS | 1 |
|  |  |  | QRS duration in lead V2 >113ms | 1 |
| Delise score |  |  | Inducible PES | 1 |
| Risk factor | Score |  |  |  |
| Spontaneous type-1 pattern | 1 |  | Kawazoe score | |
| Syncope | 1 |  | Risk factor | |
| Family history of SCD | 1 |  | Syncope | |
| Inducible PES* | 1 |  | r-J interval in lead V1 | |
|  |  |  | Syncope | |
| * In the subgroup of patients undergoing EPS  only |  |  | QRS duration in V6 | |
|  |  |  | Tp-e dispersion | |

**Supplementary Table 5** – Individual risk scores

**Supplementary Table 6** – Reported C-statistics from the model development and external validation analyses.

| **Reference** | **Analysis type** | **For external validations, reference for model development study if different from listed study** | **Type of study** | **Modelling method** | **Reported c-statistic (95% CI)** |
| --- | --- | --- | --- | --- | --- |
| (1) Honarbakhh, 2021 | D+EV |  | Existing registry | Cox regression | 0.88 (0.81-0.94)* |
| (2) Kawada, 2018 | EV | Shanghai score | Retrospective cohort |  | 0.76 (0.70-0.82) |
| (3) Delise, 2010 | D |  | Retrospective cohort | Cox regression | 0.77 (0.71-0.82) |
| (4) Sieira , 2017 | D+EV |  | Existing registry | Cox regression | 0.81 (0.75-0.87)* |
| (5) Subramanian, 2019 | D+EV |  | Retrospective cohort | Logistic regression | 0.99 (0.99-0.99)* |
|  | EV | Kawazoe model | Retrospective cohort |  | 0.83 (0.74-0.91) |
| (6) Lee, 2022 | EV | Sieira model | Retrospective cohort |  | 0.81 (0.75-0.87) |
|  | EV | Delise model | Retrospective cohort |  | 0.66 (0.60-0.73) |
|  | EV | Shanghai score | Retrospective cohort |  | 0.70 (0.67-0.78) |
|  | EV | Okamura model | Retrospective cohort |  | 0.67 (0.60-0.73) |
|  | EV | BRUGAGA-RISK score | Retrospective cohort |  | 0.60 (0.52-0.68) |
|  | EV | Letsas model | Retrospective cohort |  | 0.66 (0.59-0.72) |
| (7) Letsas, 2019 | D |  | Existing registry | Cox regression | NR |
|  | EV | Sieira model | Existing registry |  | 0.87 (0.75-0.99) |
|  | EV | Delise model | Existing registry |  | 0.87 (0.73-1.00) |
|  | EV | Okamura model | Existing registry |  | 0.87 (0.77-0.98) |
| (8) Shinohara, 2020 | D |  | Existing registry | Cox regression | NR |
| (9) Okamura, 2015 | D |  | Retrospective cohort | Cox regression | NR |
| (10) Kawazoe, 2016 | D+IV |  | Case-control | Cox regression | 0.85 (0.79-0.92) |
| (11) Rattanawong, 2023 | D+EV |  | Retrospective cohort | Logistic regression | 0.97 (0.94–0.99)* |
|  | EV | Shanghai score | Retrospective cohort |  | 0.71 (0.53–0.88) |
|  | EV | BRUGAGA-RISK score | Retrospective cohort |  | 0.72 (0.54–0.90) |
|  | EV | Sieira model | Retrospective cohort |  | 0.82 (0.69–0.95) |
| (12) Probst, 2021 | EV | Shanghai score | Retrospective cohort |  | 0.73 (0.67–0.79) |
|  | EV | Sieira model | Retrospective cohort |  | 0.71 (0.61–0.81) |
| (13) Rodríguez-Mañero, 2022 | EV | Shanghai score | Retrospective cohort |  | 0.80 (0.75-0.85) |
|  | EV | Sieira model | Retrospective cohort |  | 0.81 (0.76-0.86) |
|  | EV | Delise model | Retrospective cohort |  | 0.77 (0.71-0.83) |
| (14) Chow, 2021 | EV | Sieira model | Case-control |  | 0.58 (0.46-0.70) |
| (15) Delinière, 2019 | D |  | Case-control | Cox regression | NR |
| (16) Kamakura, 2024 | EV | PAT score | Retrospective cohort |  | 0.71 (0.65-0.77) |
|  | EV | BRUGAGA-RISK score | Retrospective cohort |  | 0.60 (0.52-0.80) |
|  | EV | Shanghai score | Retrospective cohort |  | 0.83 (0.78-0.88) |
|  | EV | Sieira model | Retrospective cohort |  | 0.82 (0.77-0.87) |

* In studies in which a model was both developed and externally validated, only AUC of the validation cohort is reported.

Abbreviations as in Table 1.

**Supplementary Figure 1** – Overall PROBAST risk of bias (A), and applicability (B) assessment.


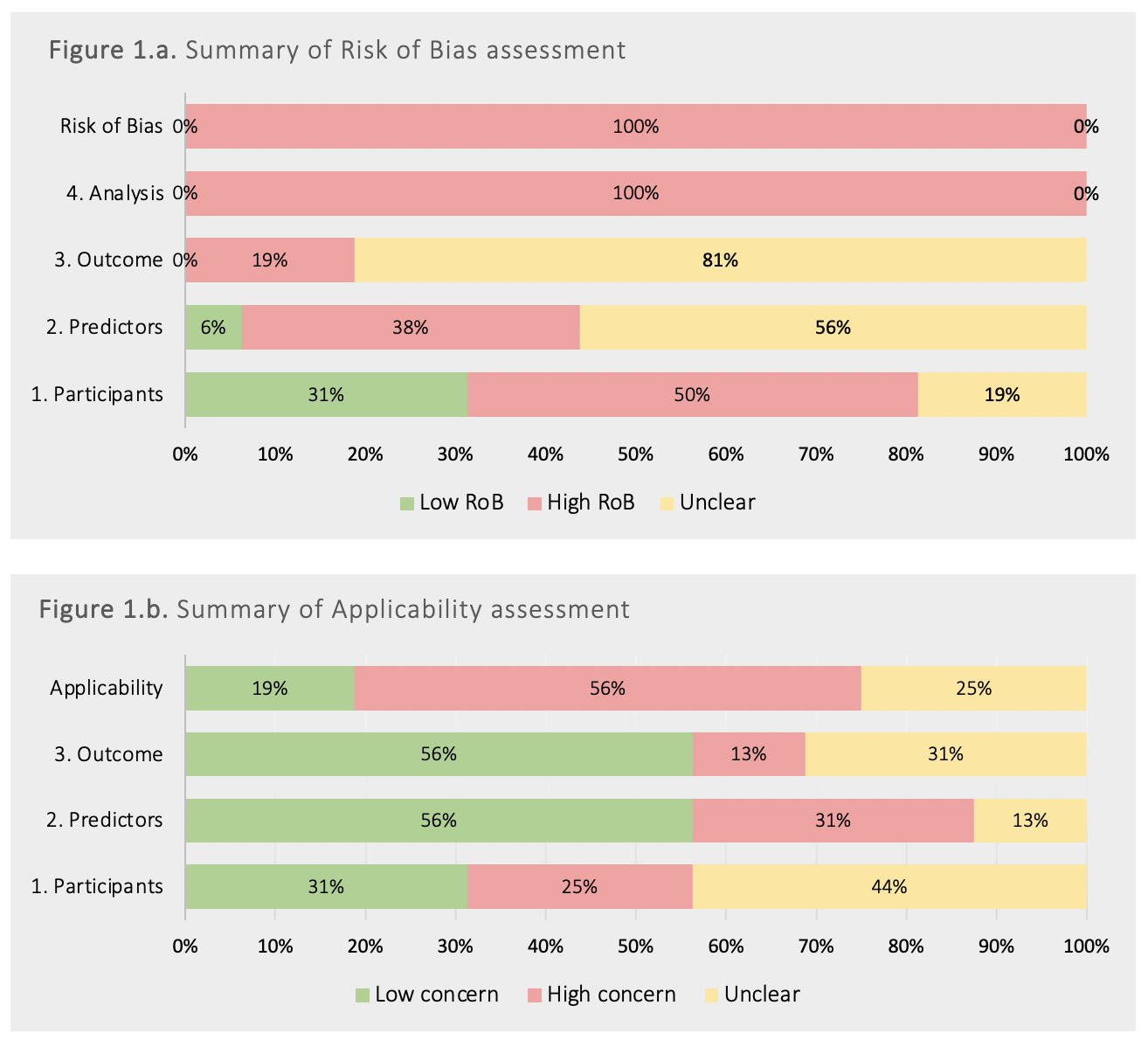

Supplement: euaf091_Supplementary_Data [file euaf091_supplementary_data.zip › Supplementary appendix_27_10.docx]
